# A newly developed oxime K203 is the most effective reactivator of tabun-inhibited acetylcholinesterase

**DOI:** 10.1186/s40360-018-0196-3

**Published:** 2018-02-21

**Authors:** Kamil Kuca, Kamil Musilek, Daniel Jun, Jana Zdarova-Karasova, Eugenie Nepovimova, Ondrej Soukup, Martina Hrabinova, John Mikler, Tanos C. C. Franca, Elaine F. F. Da Cunha, Alexandre A. De Castro, Martin Valis, Teodorico C. Ramalho

**Affiliations:** 1 Biomedical Research Center, University Hospital Hradec Kralove, Hradec Kralove, Czech Republic; 2 Department of Chemistry, Faculty of Science, University of Hradec Kralove, Hradec Kralove, Czech Republic; 3 Faculty of Military Health Sciences, University of Defence, Hradec Kralove, Czech Republic; 4 Defence Research and Development Canada - Suffield Research Centre, Department of National Defence, Suffield, Alberta, Canada; 5 Center for Basic and Applied Research, Faculty of Informatics and Management, University of Hradec Kralove, Hradec Kralove, Czech Republic; 6 Department of Chemical Engineering, Military Institute of Engineering, Rio de Janeiro, RJ 22290-270 Brazil; 7 Department of Chemistry, Federal University of Lavras, Lavras/MG, Brazil; 8 Neurology Clinic, University Hospital Hradec Kralove, Hradec Kralove, Czech Republic

**Keywords:** Antidotes, Chemical warfare agents, Poisoning, Treatment, Reactivator, Oxime

## Abstract

**Background:**

Based on in vitro and in vivo rat experiments, the newly developed acetylcholinesterase (AChE) reactivator, K203, appears to be much more effective in the treatment of tabun poisonings than currently fielded oximes.

**Methods:**

To determine if this reactivating efficacy would extend to humans, studies were conducted in vitro using human brain homogenate as the source of AChE. The efficacy of K203 was compared with commercially available oximes; pralidoxime, obidoxime and asoxime (HI-6).

**Results:**

Reactivation studies showed that K203 was the most effective reactivator with a second order kinetic constant (*k*_r_) of 2142 min^− 1^. M^− 1^, which was 51 times higher than that obtained for obidoxime (*k*_r_ = 42 min^− 1^. M^− 1^). Both pralidoxime and asoxime (HI-6) failed to significantly reactivate tabun-inhibited human AChE.

**Discussion:**

According to these results and previous studies, using K203, it appears that oxime K203 is the most effective reactivator of tabun-inhibited cholinesterase in several species including humans and should be considered as a possible medical countermeasure to tabun exposure.

## Background

**Fig. 1:**
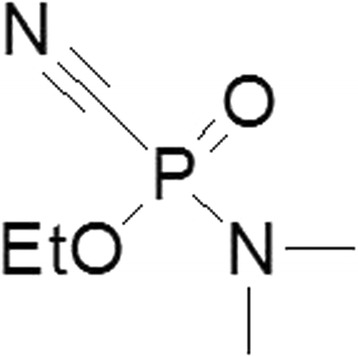
Chemical structure of tabun

Organophosphorus nerve agents comprise a group of the most toxic chemicals ever synthesized. [1] There is currently a lack of sufficiently effective antidotes against the entire spectrum of these compounds [2, 3]. Tabun (Fig. 1), is considered one of the most toxic compounds within this group; especially considering that there are very few fielded cholinesterase-reactivators able to treat tabun intoxications [4–6]. Tabun’s extraordinary toxicity and resistance to current medical countermeasures appear to be the result of inhibition of acetylcholinesterase (AChE), which is difficult to reactivate due to the presence of a lone electron pair on the amidic group [7]. This novel enzyme inhibition mechanism was presented by Ekstrom et al. using a theoretical binding site model [8]. However, from a treatment point of view, if the efficacy of cholinesterase-reactivation directly correlates to the effectiveness of treatment, there are several reactivators with low to moderate potency to treat tabun intoxications.

Among these reactivators trimedoxime and obidoxime, symmetrical bispyridinium bis-oximes, are considered the best of the commercially available acetylcholinesterase (AChE) reactivators [4, 9, 10]. Unfortunately their use has been associated with adverse effects [11, 12]. Consequently novel reactivators with higher reactivation activity and lower toxicity should be developed [13]. In 2003, two new oximes aimed at the reactivation of tabun-intoxications, K027 and K048 were developed [14, 15]. They differ from the chemical structure of trimedoxime and obidoxime, which are symmetric compounds, and a carbamoyl group was introduced into their structure to decrease their toxicity. Their in vitro and in vivo properties were shown to be promising [5, 16–21]. Two additional oximes were synthesized – K074 and K075 – differing from trimedoxime and obidoxime by the linker between the two heteroarenium rings [22, 23]. Although these oximes achieved higher reactivation potency, they were more toxic than the original compounds [24, 25].

**Fig. 2:**
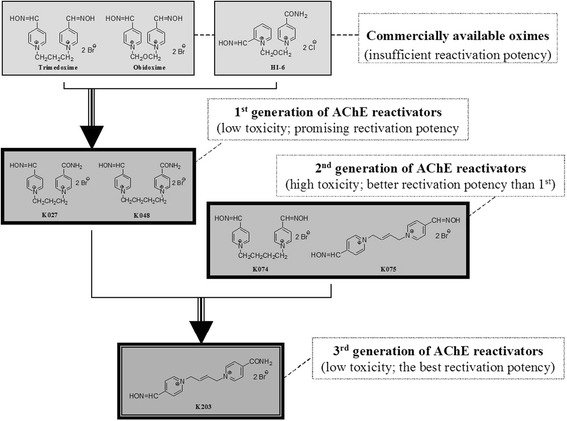
Developmental strategy of the AChE reactivator K203

Recently a new low-toxic oxime with high reactivation potency was developed, K203 [26, 27] (Fig. 2). When compared with other oximes K203 has been shown to have the best reactivation activity and toxicity. All previous studies with K203 have been conducted in rodents and therefore we were interested in determining its in vitro reactivation of human brain AChE. As is known, differences in reactivation are observed depending on the species used [22, 23, 28, 29]. This raises the intriguing question of how to improve the action of drugs. Firstly, one way is to identify structural and electronic determinants of drug sensitivity by mean of experimental data as well as theoretical calculations, which in turn provide important clues about the active site of the molecular targets and its interaction with inhibitors. Thus, in the current work, to rationalize the electronic and steric features that modulate the oxime action, molecular modeling techniques were employed. In this line, the the current study demonstrates the efficacy of K203 reactivation of tabun-inhibited AChE in human brain homogenate. Commercially available pralidoxime, obidoxime, asoxime (HI-6) were used in this study as the standards for comparison.

## Methods

### Chemicals

**Fig. 3:**
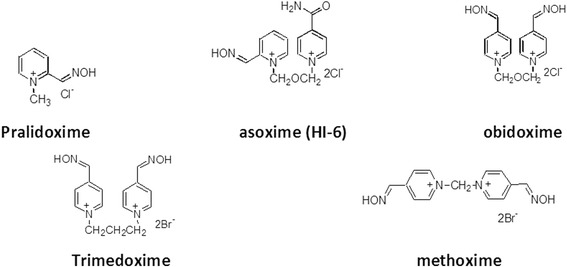
Currently commercially available oximes

K203 ((*E*)-1-(4-carbamoylpyridinium)-4-(4-hydroxyiminomethylpyridinium)-but-2-ene dibromide) was prepared at the Toxicology Department according to the published method [26]. The structure of K203 and structure of oximes tested for comparison (pralidoxime, obidoxime, asoxime (HI-6)) are shown in Fig. 3. Purity of all tested reactivators was measured using TLC (DC-Alufolien Cellulose F; Merck, Germany; mobile phase BuOH-CH_3_COOH-H_2_O 5–1-2; detection by solution of Dragendorff reagent), HPLC (P200 gradient pump Spectra-Physics Analytical, Fremont, USA; a 7125 injection valve – 10 ul loop, Rheodyne, Cotati, USA; an UV1000 detector, Spectra-Physics Analytical, Fremont, USA) and melting point determination (Micro heating stage PHMK 05; VEB Kombinat Nagema, Radebeul, Germany) [30, 31].

Tabun (GA; *O*-ethyl-*N,N*-dimethyl phosphoramidocyanidate) was obtained from the Military Facility Brno and was shown to have a purity of at least 97% purity. All other chemicals used were of reagent grade (Sigma-Aldrich, Czech Republic).

### Source of human brain cholinesterase

The nucleus caudatus was obtained from autopsy patient material (male) aged 68 and 70, who died by accident (Department of Forensic Medicine, Faculty of Medicine in Hradec Kralove, Charles University In Prague). The brain parts were directly frozen. Ex tempore, 1–2 g of defrosted human brain tissue macroscopically free from white matter was taken and homogenized by Ultra-Turrax apparatus in saline solution (1/10, wet weight/volume of 0.9% saline solution). Experiments were approved in compliance with relevant laws and institutional guidelines and were approved by the Ethics Committee of the Faculty of Military Health Sciences in Hradec Kralove (Czech Republic).

### Cholinesterase assay

Cholinesterase activity was measured using the procedures previously published by Kuca and Kassa [16]. Briefly, reactivation efficacy of the oximes was evaluated in an in vitro model of brain cholinesterases inhibited by tabun using the standard reactivation method with electrometric instrumentation. The human brain homogenate (0.5 ml) was mixed with 0.01 μM isopropanol tabun solution (0.5 ml) and then incubated for 30 min at room temperature to reach approximately 95% AChE inhibition. Tabun-inhibited AChE was incubated for 10 min with an oxime solution (1 ml) at various oxime concentrations. Following incubation, 3 M sodium chloride solution (2.5 ml) was added, along with distilled water, to a constant volume (23 ml). Finally, 0.02 M acetylcholine iodide (2 ml) was added and the enzyme activity was measured titrimetrically at pH 7.6 and 25 °C on an Autotitrator RTS 822 (Radiometer, Denmark).

### Computational details

#### Docking procedure

Firstly, the molecular docking technique was performed using the reactivators K203 and Obidoxime to perform the docking methodology with the non-aged form of *Mus musculus* Acetylcholinesterase (*Mm*AChE) inhibited by tabun (PDB code 3DL4; resolution = 2.50 Å) [32], and with the human enzyme, also inhibited by this nerve agent, being a model of *Hss*AChE already used in other works [33–35]. The oximes structures were constructed by using the PC Spartan Pro® software [36], with a subsequent optimization at the DFT level, with the Gaussian 09 package [37], using B3LYP density functional and 6-31 g (d, p) basis set. The partial charges of the atoms were elucidated via the Chelpg method [34]. The ligands were docked within the crystallographic structure of both AChE species by employing the Molegro Virtual Docker software (MVD®) [38], considering the same procedures previously employed [39–41]. For the docking procedure, within a radius of 8 Å, water molecules and amino acid residues were considered as flexible. Due to the essence of the molecular docking methodology, the calculations were performed giving rise to 50 different conformations (poses) for the reactivators. The most significant conformation for each ligand was chosen taking into account the most promising interaction energy within the enzyme active site, always searching for appropriate accommodations in the site, beyond the potent conformations for the reactivation mechanism.

The MolDock scoring function employed in the MVD software has its origin on the piecewise linear potential (PLP), a reduced potential whose settings are adjusted to enzyme-ligand complexes [42]. The docking scoring function values, E_score_, are defined by Eq. 1:

1 $$ {\boldsymbol{E}}_{\boldsymbol{score}}={\boldsymbol{E}}_{\boldsymbol{inter}}+{\boldsymbol{E}}_{\boldsymbol{intra}} $$

Where:

2 $$ {\boldsymbol{E}}_{\boldsymbol{E}\boldsymbol{inter}}=\sum \limits_{\boldsymbol{i}\boldsymbol{\varepsilon } \boldsymbol{ligand}}\sum \limits_{\boldsymbol{j}\boldsymbol{\varepsilon } \boldsymbol{protein}}\left[{\boldsymbol{E}}_{\boldsymbol{PLP}}\left({\boldsymbol{r}}_{\boldsymbol{i}\boldsymbol{j}}\right)+332.0\ \frac{\boldsymbol{qiqj}}{4{\boldsymbol{r}}_{\boldsymbol{i}\boldsymbol{j}}^2}\ \right]\kern0.75em $$

E_PLP_ stands for “piecewise linear potential”, which consists in the use of two different parameter sets, as described as follows: one employed for approach of the steric term among atoms (Van der Waals), and the other for the hydrogen bonding. The second term is associated to electrostatic interactions in relation to overloaded atoms. It consists of a Coulomb potential, presenting a dielectric constant dependent on the range (D(r) = 4r). The numeric amount of 332.0 is important, given that the electrostatic energy unit can be provided in kilocalories per molecule [38]. E_intra_ is associated to the inner energy of the ligand:

3 $$ {\boldsymbol{E}}_{\boldsymbol{i}\boldsymbol{ntra}}=\sum \limits_{\boldsymbol{i}\boldsymbol{\varepsilon } \boldsymbol{ligand}}\sum \limits_{\boldsymbol{j}\boldsymbol{\varepsilon } \boldsymbol{ligand}}{\boldsymbol{E}}_{\boldsymbol{PLP}}\left({\boldsymbol{r}}_{\boldsymbol{i}\boldsymbol{j}}\right)+\sum \limits_{\boldsymbol{flexiblebonds}}\boldsymbol{A}\left[1-\cos \left(\boldsymbol{m}.\boldsymbol{\theta} -{\boldsymbol{\theta}}_0\right)\right]+{\boldsymbol{E}}_{\boldsymbol{clash}} $$

The first portion of the equation (double addition) is related to all pairs of atoms in the ligand, ruling out those that are linked by two bonds. The second term qualifies the torsional energy, where θ is the torsional angle of the bond. If several torsions are capable of being resolved, each torsional energy is important and an average in relation to them is employed. The last measure, E_clash_, attributes a penalty equals to 1000 in the situation of distances less than 2.0 Å among heavy atoms, not considering impracticable ligand conformations [38]. The search algorithm in MVD combines the differential evolution optimization method with a cavity forecast algorithm, throughout the search process, thus allowing a rapid and precise recognition of potential binding modes (poses) [38, 43, 44].

### Reaction mechanism and QM/MM methodology

From the structures selected with the docking methodology, the hybrid QM/MM was carried out to evaluate the energetic barrier (activation energy) for the mechanism in the reactivation pathway of the rat and human AChE inhibited by tabun. QM/MM techniques allow the modeling of larger systems, like reactions within enzymes, by combining the electronic degrees of a quantum chemical approach with the MM methods, increasing performance and decreasing computational demand [45]. Thus, in this study, hybrid quantum and molecular mechanics (QM/MM) associated with molecular docking methods have been carried out to determine the reactivation reaction pathway for the inhibited AChE, using different reactivators. Actually, this theoretical strategy has been previously applied in other occasions [35, 46–48]. With the purpose of getting more accurate results, capturing electronic effects, QM calculations were performed at the density functional theory (DFT) level with the Gaussian 09 package [37]. DFT techniques have shown a significant performance for large systems, such as biomolecules [49–51]. This relationship between functional and basis sets has been tested for similar systems. [44, 52]

The QM systems were constituted of amino acid residues, water molecules, reactivators and Ser203-tabun complex, for both AChE species. In the reaction mechanism simulation, all transition states were calculated and characterized identifying imaginary frequencies [53, 54]. Each system was fully optimized at DFT level, with conjugate gradient and quasi-Newton-Raphson algorithms. The final geometries were obtained with B3LYP functional density [43], using 6-31 g (d, p) basis set.

## Results

**Fig. 4:**
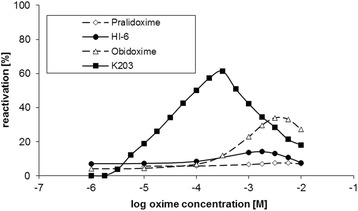
The effectiveness of oximes at varying concentrations to reactivate tabun-inhibited human cholinesterase. Cholinesterase activity measured in human brain homogenate is shown as a percentage of control activity. Tabun was incubated with brain homogenate for 30 min prior to the addition of the oxime. Reactivation was carried out for 10 min. All experiments were carried out at pH 7.6 and 25 °C. Each point represents the mean±SEM for 2 measurements

In vitro results obtained in this study are summarized in Table 1 and Fig. 4. The reactivation process is characterized by several constants. Constant *K*_R_ represents the affinity of the AChE reactivator to the inhibited enzyme. *k*_R_ is the first order kinetic constant characterizing the splitting of the bond between enzyme and inhibitor. And finally, *k*_r_ is the second order kinetic constant characterizing the entire reactivation process.

**Table 1:** Tabun-inhibited acetylcholinesterase reactivation constants

	*HUMAN*			*RAT*	
Constants	*K*_R_ [μM]	*k*_R_ [min^−1^]	*k*_r_ [min^− 1^. M^− 1^]	*k*_r_^c^ [min^− 1^. M^− 1^]	Species Difference ratio
Oxime					
Pralidoxime	- ^a^	- ^a^	- ^a^	10	- ^b^
Obidoxime	1412	0.060	42	6250	149
Asoxime (HI-6)	- ^a^	- ^a^	- ^a^	1111	- ^b^
K203	56	0.120	2142	16,000	7.5

^a, b^Unable to estimate the appropriate constants due to lack of reactivated AChE by this oxime

^c^Rat brain homogenate results published by Musilek et al. J. Med. Chem. (2007)

From the results obtained, only obidoxime and K203 were able to reactivate tabun-inhibited human brain cholinesterases. When they were directly compared, K203 surpassed obidoxime in all parameters including a 25-times higher affinity towards the inhibited enzyme. K203 was also able to disrupt the inhibitor-enzyme complex at twice the rate of obidoxime. Based on these two parameters the overall second order kinetic constant characterizing the whole reactivation process (*k*_r_) favored K203 for reactivation of tabun-inhibited AChE. If the reactivation curves were compared, K203 surpasses all other reactivators tested in this study. K203 reactivated over 60% of cholinesterase activity at human attainable concentrations. Obidoxime was the second most efficient oxime with 33% reactivation, but at concentrations between 10^− 2^ and 10^− 3^ M that are supposed to be not attainable in plasma after i.v. or i.m. administration. Other oximes did not appreciably reactivate tabun-inhibited human AChE.

## Discussion

This study is the first to compare the effectiveness of four oximes in reactivating tabun-inhibited acetylcholinesterase (AChE) in human brain homogenate. Only five oximes are currently clinicaly available worldwide for the treatment of organophosphorus nerve agent or pesticide poisoning, pralidoxime, trimedoxime, obidoxime, methoxime and asoxime (HI-6). Asoxime (HI-6) has been the most extensively investigated as a broad-spectrum antidote but has been shown to be a very weak reactivator of tabun-inhibited AChE in several rodent species [9, 10, 22, 23, 55, 56]. The ineffectiveness of asoxime (HI-6) has also been reported in in vivo and in vitro studies conducted in rats [26, 57]. Of the commercially available oximes, only trimedoxime and obidoxime are able to reactivate tabun-inhibited AChE, however, their reactivation potency is less than ideal [4, 5]. K203 has been previously shown to be the most effective reactivator of tabun-inhibited AChE in both in vitro and in vivo rodent studies [57]. This study was the first to evaluate the efficacy of K203 on real human brain AChE and as was observed in the rodent study, K203 is more effective in reactivating tabun-inhibited enzyme than obidoxime. At therapeutic concentrations, K203 was able to reactivate over 60% of tabun-inhibited AChE, while obidoxime reactivated only 33% of its activity at supra-pharmacological doses (Fig. 4). When the overall reaction rate constant (*k*_*r*_) of human tissue is compared (Table 1), the constant for K203 was 51-times higher than that of obidoxime but only 2.5-times higher when the rat tissue was tested [26]. However when *k*_*r*_ ratios are compared between human and rat enzymes, obidoxime results decrease 149-times while K203 decreases only 7.5-times. These results suggest that a direct correlation in oxime-mediated reactivation between species does not exist and that comparative studies in one species may not truly reflect the reactivation effects in humans.

**Fig. 5:**
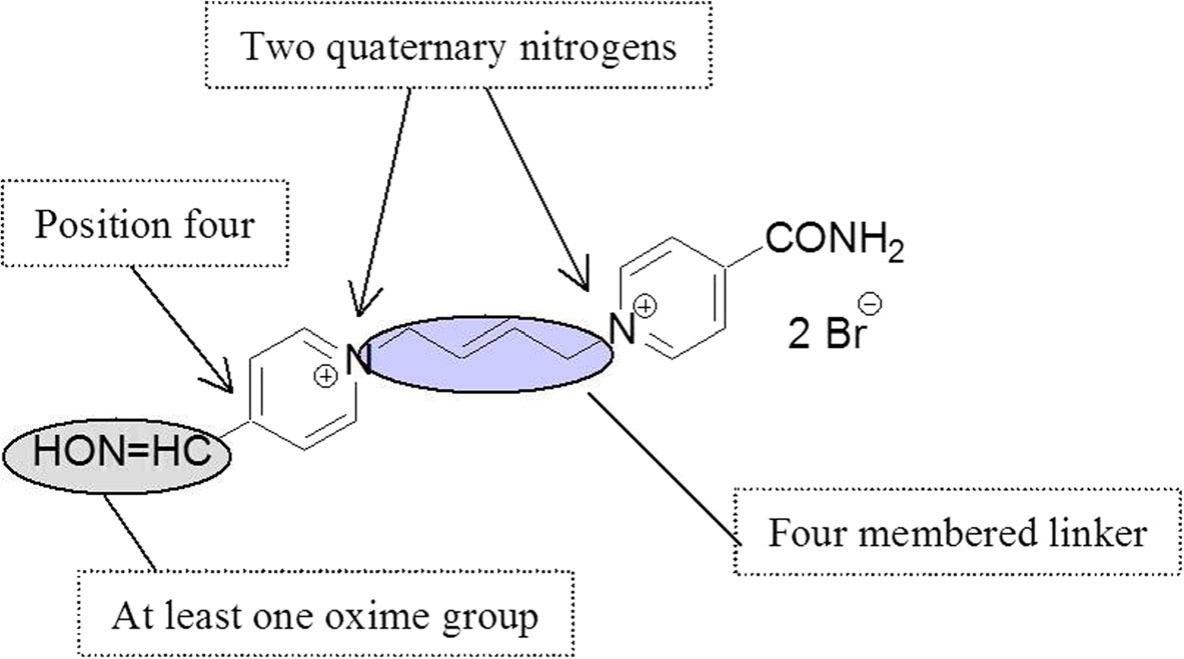
Structural requirements for reactivators of tabun-inhibited AChE

The better reactivation efficacy of K203 was expected based on structure-activity relationship requirements [4, 58]. K203 meets four main requirements; two quaternary pyridinium rings, one nucleophilic oxime group, oxime at position four on the pyridinium ring and four carbon linkages between pyridinium rings (Fig. 5). Hundreds of compounds have been synthesized over the last decade to develop an effective reactivator of tabun-inhibited AChE. Of these compounds, K027, K048, K074 and K075 have yielded promising results against other organophosphorus compounds and are currently being evaluated by several laboratories worldwide [5, 19, 21, 59–64]. However, K203 appears to be the most effective reactivator following tabun exposure and it has also been reported to have a lower acute toxicity than trimedoxime and obidoxime [26]. The efficacy and toxicity profile of K203 supports the continued efforts in developing this compound as a medical countermeasure against.

### Molecular affinity: Docking studies

At the first scenario, molecular docking calculations have been used in order to look into the association/affinity between oxime and inhibited AChE. A cavity forecast algorithm, which is based on a 3D box was employed to bring forth binding sites in the enzymes, and for this, the Molegro Virtual Docker program was quite helpful. [38] The oximes were docked in a cavity whose volume was 115,200 Å [3] in *Mm*AChE and 282,112 Å [3] in *Hss*AChE. The respective energy values acquired from the oximes-AChE interactions were determined to have a better understanding the binding modes of each ligand, thus investigating the structural aspects that modulate the biological activity in the enzyme reactivation. Table 2 shows the intermolecular interaction energy values of the most appropriate pose of K203, selected for the subsequent mechanistic studies in both enzymes. By observing Table 2, it is easy to notice that K203 interacted better with the human enzyme, *Hss*AChE, with an intermolecular interaction energy value of − 101.94 kcal.mol^− 1^, and an energy difference of 12.16 kcal.mol^− 1^ in relation to the rat enzyme, *Mm*AChE. K203 has shown itself to have a high stability in the *Mm*AChE, experimenting hydrogen bonds with two water molecules as well as two amino acid residues, Thr238 and Gly234. Interestingly the fact that this oxime presented much more hydrogen bonds in the *Hss*AChE active site, where it also presented a good intermolecular interaction energy, − 89.78 kcal.mol^− 1^. In the human enzyme, K203 showed several interactions with amino acid residues and water, more precisely, six water molecules and two amino acid residues, Tyr120 and Gly117. Besides those interactions in the *Hss*AChE active site, K203 showed a better energy value when docked in the *Mm*AChE active site, and with this event, one can suppose that there are other important features that assist the good stabilization of this oxime in the enzyme active site, such as structural aspects, i.e., *Mm*AChE interacts better with K203 in relation to *Hss*AChE.

**Table 2:** Intermolecular interaction energy (ΔE) values and hydrogen bonds between K203 and both AChE species

AChE	Interaction Energy (kcal.mol^− 1^)	H-Bonds	H-bond Strength (kcal.mol^− 1^)
Human	−89.78	H_2_O	− 4.92
		H_2_O	−2.50
		H_2_O	−1.47
		H_2_O	−1.39
		H_2_O	−4.73
		H_2_O	−4.79
		Tyr120	−0.66
		Gly117	−1.34
Rat	−101.94	H_2_O	−3.89
		H_2_O	−2.41
		Thr238	−6.12
		Gly234	−5.00

**Fig. 6:**
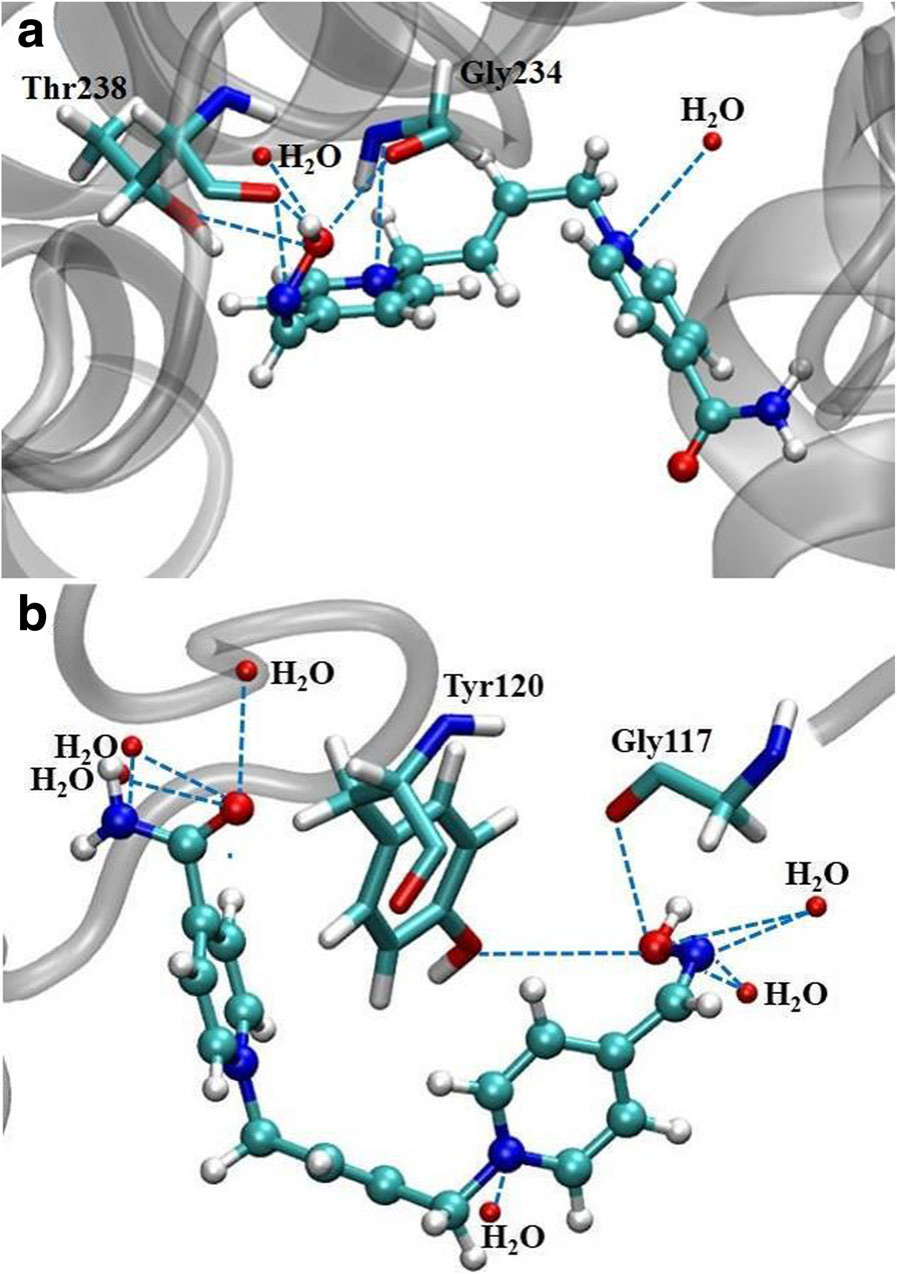
Interactions performed by K203 in the *Mm*AChE (**a**) and *Hss*AChE (**b**) active site

The docking methodology provides a good start to understand the performance of these oximes in the inhibited AChE active site. It is important to keep in mind that diverse interactions are responsible for the better accommodation and affinity of these reactivators within the active site. Among these interactions, the hydrogen bonds are the most common (H-bonds), but it is noteworthy to cite electrostatic interactions as well as hydrophobic interactions, which could even have a great contribution to the conformation and orientation adopted by the reactivators in the active site. The H-bonds performed by this oxime, in both enzymes, are shown in Fig. 6.

Both enzymes (*Mm*AChE and *Hss*AChE) were evaluated in order to have a deeper understanding of their main aspects and hydrophobic and electrostatic parameters. All these features, including the flexibility effect on the residues in a significant radius of the active site, contributed to simulate the interaction of these reactivators with the inhibited enzymes, producing a good starting point for the reaction mechanism calculations. The theoretical methodologies employed here are quite important in the design of new drugs. Other docking calculations were also carried out with Obidoxime. These results are presented in Table 3.

**Table 3:** Intermolecular interaction energy (ΔE) values and hydrogen bonds between Obidoxime and both AChE species

AChE	Interaction Energy (kcal.mol^−1^)	H-Bonds	H-bond Strength (kcal.mol^− 1^)
Human	−107.98	H_2_O	−2.43
		H_2_O	−4.17
		H_2_O	−2.50
		H_2_O	−5.00
		Ser294	−2.24
		Glu281	−2.50
		Gly117	−1.69
Rat	−91.88	H_2_O	−1.18
		H_2_O	−3.46
		H_2_O	−5.00
		Thr238	−7.52
		Gly234	−1.18
		Glu313	−2.24
		Asn533	−0.72
		Gln369	−0.96

**Fig. 7:**
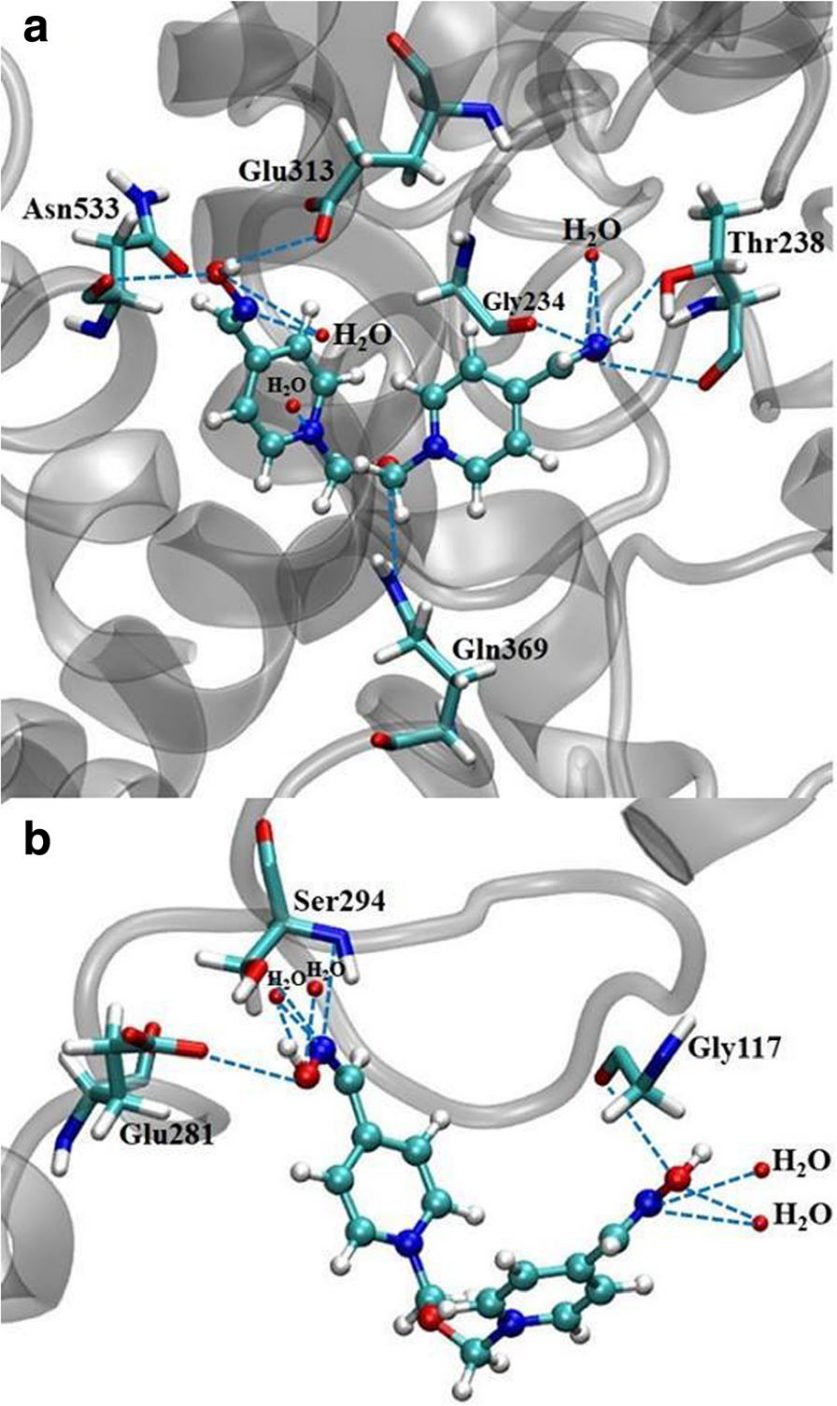
Interactions performed by Obidoxime in the *Mm*AChE (**a**) and *Hss*AChE (**b**) active site

From the theoretical calculations, the docking results for Obidoxime are shown in Table 3. Those findings indicate that Obidoxime presented higher stability when docked in the *Hss*AChE active site, with an intermolecular interaction energy of − 107.98 kcal.mol^− 1^. On the other hand, this oxime also interacted very favorably with the rat enzyme, with an energy value of − 91.88 kcal.mol^− 1^. For Obidoxime, the energy difference was 16.10 kcal.mol^− 1^ between both enzymes. Obidoxime performed several hydrogen bonds in the *Hss*AChE active site, being four water molecules and three amino acid residues, Ser294, Glu281 and Gly117, which are important for the stabilization of this reactivator in the site. Meanwhile, this oxime also presented many interactions in the *Mm*AChE active site, being three water molecules and many amino acid residues (Thr238, Gly234, Glu313, Asn533 and Gln369), thus showing the great affinity of the rat enzyme with this antidote. These interactions are shown in Fig. 7. Our docking calculations are in good agreement to *K*_R_ values, which represent the affinity of the AChE reactivator to the inhibited enzyme.

### Chemical reactivity: Quantum studies in the rat and human AChE active site

For the reactivation process, it is necessary to take into account the steric and electronic effects in the reaction as well as the preferential binding modes of the reactivators. In this context, the QM/MM techniques could be employed for the understanding of the binding modes between the oximes K203 and Obidoxime in the AChE active site, for both human and rat species. During the reactivation mechanism simulation, the energetic barriers for the reaction process (activation energy) were obtained and the values described in Table 4.

**Table 4:** Relative activation energy values (**∆∆E**^**#**^) obtained for the reaction mechanism in the reactivation process and experimental results

Oxime/Enzyme	ΔΔE^#^	*k*_r_ [min^−1^. M^−1^]
*K203*		
Rat	0.00	16,000
Human	38.81	2142
*Obidoxime*		
Rat	0.00	6250
Human	54.42	42

According to Table 4, the oximes K203 and Obidoxime are most reactive in the rat enzyme than in the human AChE. These quantum theoretical results corroborate our experimental rate constant (k_r_) values very well. K203 has shown itself to be very efficient in reactivating the inhibited *Mm*AChE, revealing an energetic barrier of 38.81 kcal.mol^− 1^ lower than the value obtained for the human enzyme, which is in a good agreement with the experimental results, wherein k_r_ is 16,000 min^− 1^.M^− 1^ for the rat enzyme. This fact leads us to believe that the transition state is better stabilized in the reactivation pathway of *Mm*AChE than *Hss*AChE, allowing the oxime to interact stronger with the nerve agent.

This trend also corroborates to the intermolecular interaction energies from molecular docking calculations (Table 2), which have shown the best interaction and stability of K203 in *Mm*AChE**.** From the experimental side (Table 1), there is good agreement between binding constant (*K*_R_) and ΔE values. It should be kept in mind, however, that the mechanistic studies with Obidoxime are also in a good agreement with the experimental results. By observing the k_r_ values for the oxime mediated reactivation, the difference between the rat and human enzyme is significant, and as expected, the reaction mechanism simulation has also shown this result. Obidoxime interacted much more in the rat enzyme, with a large difference between the values found for the energetic barrier in the reaction pathway, being 54.42 kcal.mol^− 1^ in relation to *Hss*AChE. From the docking results, in contrast to K203, Obidoxime showed a slightly better interaction energy value for *Hss*AChE, but during the simulation, there must be intermediate species which are better stabilized, thus leading to lower global activation energy during the full process.

## Conclusions

We have applied computational chemistry methods in order to evaluate the high reactivity of the oximes K203 and Obidoxime for different AChE species. Our theoretical results showed that these oximes are more effective towards the rat enzyme. The reactivation process for *Mm*AChE takes place more easily by both oximes investigated. This result was confirmed by DFT calculations, being the lowest activation energy barrier for the rat enzyme, corroborating our experimental results. From molecular docking results, both reactivators are well stabilized in both AChE species, mainly due to the hydrogen bonds with the amino acid residues Ser, Glu and Gly performed in the active site.

## Abbreviations

AChE: Acetylcholinesterase
HPLC: High-performance liquid chromatography
QM/MM: Quantum and molecular mechanics
TLC: Thin-layer chromatography
